# Selective Adsorption of Quercetin by the Sol-Gel Surface Molecularly Imprinted Polymer

**DOI:** 10.3390/polym15040905

**Published:** 2023-02-11

**Authors:** Keke Zhi, Zhe Li, Han Luo, Yitong Ding, Feiyan Chen, Yongxiang Tan, Hongrui Liu

**Affiliations:** 1 Department of Engineering, China University of Petroleum-Beijing at Karamay, Karamay 834000, China; 2 State Key Laboratory of Heavy Oil Processing-Karamay Branch, Karamay 834000, China; 3 Department of Petroleum, China University of Petroleum-Beijing at Karamay, Karamay 834000, China

**Keywords:** quercetin, surface molecularly imprinted polymer, sol-gel, selective adsorption

## Abstract

Quercetin, as one of the most biologically active natural flavonoids, is widely found in various vegetables, fruits and Chinese herbs. In this work, molecularly imprinted polymer (MIP) was synthesized through surface molecular imprinting technology with sol-gel polymerization mechanism on SiO_2_ at room temperature using quercetin as the template, SiO_2_ as the supporter, 3-aminopropyltriethoxysilane (APTES) as the functional monomer, and tetraethoxysilane (TEOS) as the cross-linker. The prepared MIP was characterized via scanning electron microscope (SEM), Fourier transform infrared spectroscopy (FT-IR) and nitrogen adsorption measurements to validate its surface morphology, structure and functionality. SEM images revealed that the morphology of MIP was rough and spherical with the particle size of 260 nm larger than that of the support SiO_2_. In the FTIR spectra of MIP, the band around 1499 cm^−1^ and 2932 cm^−1^ were assigned to N−H and C-H groups, respectively. The results indicated that the imprinted polymer layers were grafted on the surface of SiO_2_ and the MIP had been successfully prepared. Since the specific surface area and pore volume of MIP were markedly higher than those of NIP and SiO_2_ and were 52.10 m^2^ g^−1^ and 0.150 cm^3^ g^−1^, respectively, it was evident that the imprinting process created corresponding imprinted cavities and porosity. The MIP for adsorbing quercetin was evaluated by static adsorption experiment. The results indicated that the adsorption equilibrium could be reached within 90 min and the maximum adsorption capacity was as high as 35.70 mg/g. The mechanism for adsorption kinetics and isotherm of MIP for quercetin was proved to conform the pseudo-second-order kinetics model (R^2^ = 0.9930) and the Freundlich isotherm model (R^2^ = 0.9999), respectively, revealing that chemical adsorption and heterogeneous surface with multilayer adsorption dominated. In contrast to non-imprinted polymer (NIP), the MIP demonstrated high selectivity and specific recognition towards quercetin whose selectivity coefficients for quercetin relative to biochanin A were 1.61. Furthermore, the adsorption capacity of MIP can be maintaining above 90% after five regeneration cycles, indicating brilliant reusability and potential application for selective adsorption of quercetin.

## 1. Introduction

Quercetin (3,3′,4′,5,7-pentahydroxyflavone), universally distributing in a large number of dietary fruits, vegetables and Chinese herbs, is the most abundant flavonoid in the plant kingdom. Extensive attention in these compounds is received to their high bioactivity including anti-oxidative [1], antiviral, anti-inflammatory [2] and antitumor activity [3]. However, quercetin typically only occurs in complicated systems and has a very low concentration. As a result, increasing the selectivity of the quercetin extraction and separation process is crucial for maintaining product quality and fully utilizing natural resources. Molecularly imprinted polymers (MIPs) possessing unique advantages in terms of its specificity and selectivity might present a desirable approach.

In a microporous three-dimensional polymer network, molecular imprinting was a technique for developing stiff and selective recognition sites that can differentiate a particular molecular target with a high degree of selectivity and affinity in preference to other structurally related compounds [4]. MIPs were sometimes referred to as manual antibodies [5] and artificial enzymes [6]. MIPs had therefore been suggested for a variety of applications, including stationary phase extractions [7,8], sensors [9,10], chromatographic separation [11,12], drug delivery [13,14] and biomimetic catalysts [15,16].

Bulk polymerization had historically been used to create MIPs [17,18,19]. However, this approach frequently led to poor binding kinetics, sluggish mass transfer and partial template removal [20,21]. Surface molecular imprinting technology, which involved creating a thin MIP layer on the surface of supporting materials, had been introduced to address these problems [22,23,24]. Surface-imprinted materials had advantages such as more accessible recognition sites, less mass transfer resistance, faster binding kinetics, and higher template removal efficiency [25,26,27]. Additionally, the presence of the support increases the surface MIPs’ physical toughness [28,29].

Several papers have been published regarding the preparation of quercetin-MIPs [30,31,32]. It was estimated that our MIP would be an available material for the selective adsorption of quercetin. The majority of reported quercetin-MIPs are synthesized utilizing functional monomers of the acrylic type by bulk thermal polymerization which requires both high temperature and nitrogen atmosphere. The reaction conditions are relatively complex and harsh [23,33,34]. In fact, the sol-gel synthetic method is known as one of the most important techniques for the formation of engineered and controlled ceramic nano-powders with a rather low process temperature. This approach also could be successfully employed to fabricate oxide, non-oxide and composite nano-powders. Furthermore, the sol-gel process promotes the preparation of the highly pure materials [35,36]. Molecularly imprinted sol-gel materials, which could be prepared at very mild conditions (room temperature was sufficient, no heating required, no need to remove oxygen by passing nitrogen) in comparison with acrylic-based MIPs has previously not been reported much. Molecularly imprinted sol-gel polymers were developed by the sol-gel method in the presence of the template molecules and the organosilane monomer in order to create stiff inorganic-organic networks by the hydrolysis and condensation of tetraethoxysilane (TEOS) [37,38,39,40,41].

In the present work, we prepared quercetin MIP by combining the merits of surface molecularly imprinting technique with the sol-gel process for the aim of selectively recognizing and binding quercetin. Quercetin was chosen as the template, SiO_2_ as the support, APTES and TEOS as the functional monomer and cross-linker, respectively. FTIR, SEM and BET surface area were used to investigate the morphology and composition of quercetin MIP. Through the binding experiments, the maximum adsorption capacity and binding kinetics of the MIP and NIP were comparatively evaluated. Using quercetin and its structural analogs, the binding selectivity and specificity of MIP for quercetin were studied. The reusability was also thoroughly discussed in order to gain a deeper understanding of MIP’s capabilities.

## 2. Experimental

### 2.1. Materials and Reagents

Quercetin was obtained from Adamas (Shanghai, China), biochanin A was obtained from Ark Pharma Scientific (Chicago, IL, USA). SiO_2_ spheres were obtained from Alfa Aesar (Shanghai, China). Methanol, acetic acid, tetraethoxysilane (TEOS), (3-aminopropyl) triethoxysilane (APTES) (98%) were obtained from Adamas (Shanghai, China). Acetone was obtained from Tianjin Zhiyuan Chemical Co., Ltd. (Tianjin, China). Water was purified to high-performance liquid chromatography (HPLC) grade by a Millipore Milli-Q gradient system.

### 2.2. Preparation of Surface MIP

The surface MIP was prepared as follows: A 250 mL flask containing 60 mL of a 33% (*w*/*w*) methanesulfonic acid aqueous solution with SiO_2_ (8.0 g) was refluxed for 8 h at 110 °C while being stirred. Following that, centrifugation was used to separate the SiO_2_ particles from the solution. The resulting SiO_2_ was then dried for 10 h at 70 °C under a vacuum before being washed with ultrapure water and methanol to a neutral pH. The activated SiO_2_ was then obtained.

Quercetin (1 mmol) was dissolved completely in 30 mL acetone. Then, 10 mmol of APTES were added under stirring for 1 h to form monomer-template complex. 500 mg of the activated SiO_2_ was mixed into the solution above under stirring for 30 min to ensure full dispersion. Finally, 4 mL of TEOS and HAc (1 mL of 1.0 mol L^−1^) were added to the suspension mixture under stirring for 40 h at room temperature to obtain particles with a high cross-linking structure. After that, Soxhlet extraction (methanol and acetic acid, *v*/*v* = 9/1) was used to wash the resulting polymer to remove quercetin. Then, neutralized with ultrapure water, washed with methanol and dried at 45 °C for 12 h in a vacuum drying oven. Finally, the MIP was obtained. The surface NIP was also prepared in the same manner without adding quercetin.

**Figure 1:**
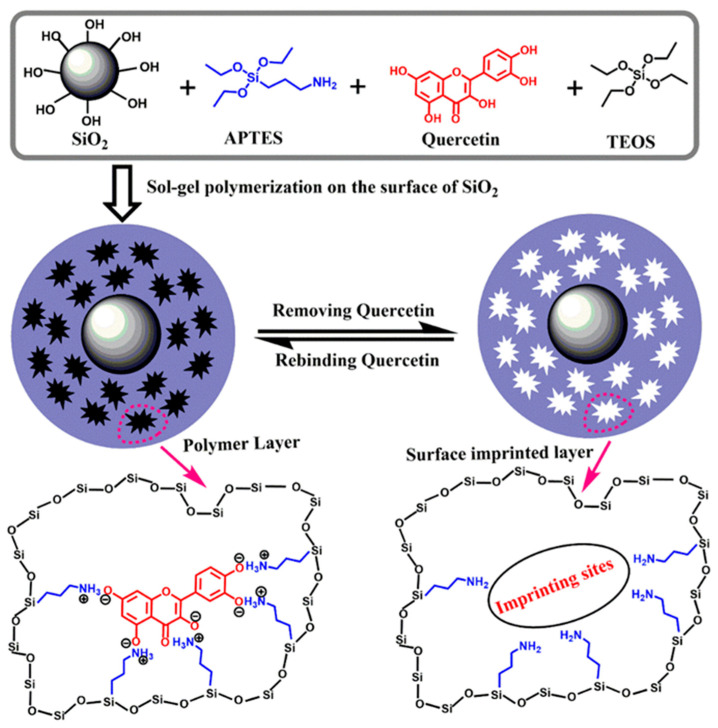
Schematic procedure of MIP preparation.

Figure 1 depicts a schematic representation of quercetin-MIP on the support of SiO_2_ overall synthesis process. Sol-gel technology and surface imprinting were coupled in our method. The template molecule quercetin (value of the dissociation constant pK_a_ about 6.6 [42]) which has five acidic hydroxyl groups—OH was weak acid. APTES was chosen as functional monomer to synthesize quercetin-MIP. It was favorable for APTES to selectively engage with quercetin via reversible acid-base ionic pair interaction since it had a basic amino group, -NH_2_. APTES served as both the functional monomer and the intermediary in the linking of SiO_2_. TEOS and APTES were then hydrolyzed and condensed in the presence of HAc catalyst to anchor the complex to the SiO_2_ surface. The rigid polymeric network was created as a result. Finally, after removing the template quercetin by acid washing to destroy the acid-base ionic pair interaction between APTES and quercetin, a huge amount of tailor-made imprinted cavities, which are complementary to the quercetin in shape, size, and chemical functionality and specifically designed for quercetin, were formed in the surface imprinted polymer layer. Thus, MIP could selectively rebind the quercetin molecule from a mixture of quercetin and the analogs.

### 2.3. Characterization

The adsorption capacity of the MIP was measured by UV-Visible spectroscopy (UV-6100PC, Mapada, Shanghai, China). To investigate surface morphology of the MIP, scanning electron microscopy (SEM) (Regulus 8100, Hitachi, Japan) was employed. Fourier transform infrared (FT-IR) spectrometer (NICOLET 5700, USA) was used to analyze the MIP composition. Nitrogen adsorption/desorption analysis was made on an automatic adsorption apparatus (Autosorb-IQ, USA) and the specific surface area (S) was calculated using the Brunauer–Emmett–Teller (BET) method.

### 2.4. Binding Experiments

To ascertain the shortest period of time needed for adsorption equilibrium, the adsorption kinetic investigation was conducted using a starting quercetin concentration of 30 mg/L. 10 mg of MIP or NIP were added to a 5 mL solution of quercetin in methanol. The mixture was incubated in a shaking bed at room temperature for intervals ranging from 5 to 120 min. Following the separation of the particles using syringe filters with a 0.22 m microporous membrane, the residual quercetin concentration was assessed using UV-visible spectroscopy at a wavelength of 372 nm. The adsorption capacity Q_t_ (mg/g) was calculated using Equation (1) below.

(1) $$Q_{t}=\frac{\left(C_{0}-C_{t}\right)V}{m}$$

where C_0_ (mg/L) and C_t_ (mg/L) were the initial and residual concentrations of quercetin, respectively. V (L) and was the volume of the solution and m (mg) was the weight of the MIP or NIP.

For the adsorption isothermal test, 10 mg of MIP or NIP was added separately to 5 mL of quercetin methanol solution with different concentrations ranging from 10 to 800 mg/L. These mixtures were shaken at 25 °C room temperature for 10 h to make sure reaching binding equilibrium. The following experimental procedures were the same as the adsorption kinetic study.

To evaluate the specific recognition property of MIP, the selective adsorption experiment was performed using biochanin A, a different flavonoid with a structure very similar to quercetin (Figure 7). A total of 10 mg MIP or NIP were added to 5 mL of 30 mg/L quercetin or biochanin A methanol solution, separately. All the mixtures were shaken for 60 min at room temperature. Moreover, imprinting factor (IF) and selectivity coefficient (α) were calculated for evaluating the recognition and selectivity of MIP towards quercetin and competitive compound. The equations were as follows.

(2) $$IF=\frac{Q_{MIP}}{Q_{NIP}}$$

(3) $$\alpha =\frac{{IF}_{T}}{{IF}_{C}}$$

where Q_MIP_ (mg/g) and Q_NIP_ (mg/g) were the capacity for adsorption of MIP and NIP for quercetin, or biochanin A at the same circumstances, respectively. IF_T_ and IF_C_ were the imprinting factor for the template molecule quercetin and the competitive compound of biochanin A.

### 2.5. Reusability of MIP

**Figure 2:**
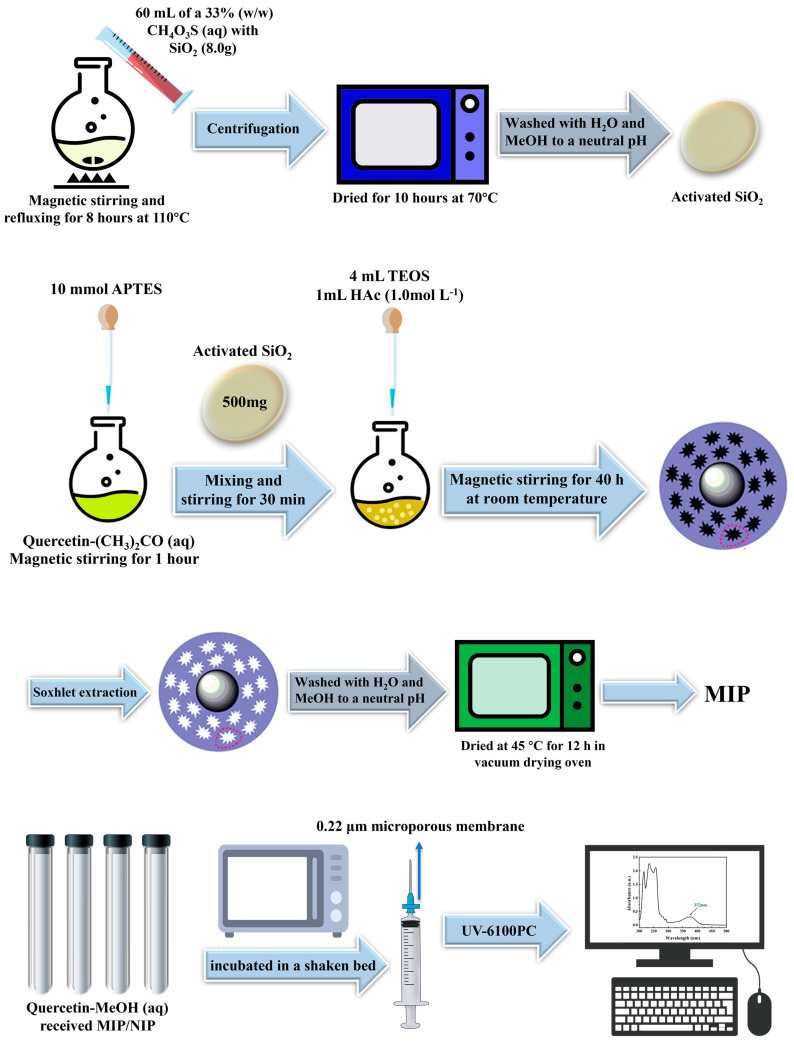
The flowchart of experimental process.

The desorption and reutilization of the MIP was investigated. During the reusability experiment, 30 mg of MIP was added to 15 mL quercetin methanol solution (800 mg/L) and was shaken for 90 min at room temperature. The mixture was centrifuged and the supernatant was passed through a syringe filter. The adsorbent MIP saturated with quercetin was washed with a mixture solution (methanol and acetic acid, *v*/*v* = 9/1) to remove quercetin, ultrapure water to neutral condition. Then, the regenerated MIP was dried and reused for the next adsorption. The adsorption–desorption experiments were repeated five times. The experimental process is depicted by a flowchart in Figure 2.

## 3. Results and Discussion

### 3.1. Characterization

#### 3.1.1. FT-IR Analysis

**Figure 3:**
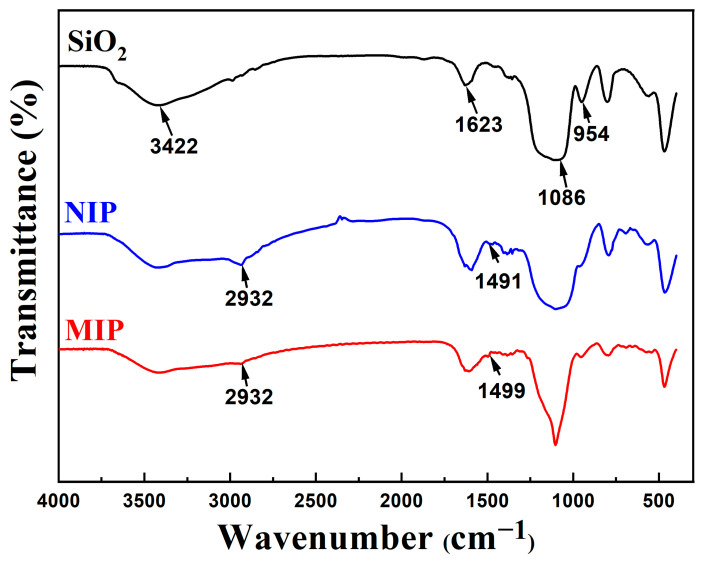
FT-IR spectra of SiO_2_ support and its corresponding NIP and MIP.

FT-IR analysis is carried out to characterize the structure details for SiO_2_ support, NIP and MIP. In the FT-IR spectra (Figure 3) of SiO_2_, the band at 3442 cm^−1^, 1623 cm^−1^, 1086 cm^−1^, and 954 cm^−1^ could be ascribed to -OH, H-O-H, Si-O-Si, and Si-O-H vibrations, respectively [43]. A characteristic feature of MIP and NIP compared with SiO_2_ was the N-H band at 1499 cm^−1^ and 1491 cm^−1^, and the C-H band around 2932 cm^−1^ [44]. It proved that APTES was successfully grafted onto the surface of SiO_2_ after imprinting.

#### 3.1.2. SEM Analysis

**Figure 4:**
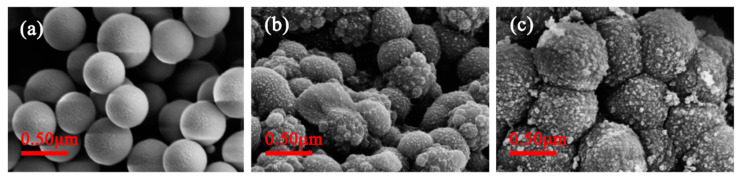
SEM images of (**a**) the SiO_2_ support, (**b**) NIP and (**c**) MIP.

The morphology of the surfaces for the SiO_2_, NIP and MIP are exemplified by the SEM in Figure 3. From the SEM images, it is obvious that all of particles were roughly spherical in shape. The SiO_2_ support (Figure 4a) shows a smooth surface. The quercetin-MIP (Figure 4c), on the other hand, exhibits extremely rough surfaces and bigger particle sizes than pure SiO_2_, which suggests the imprinted coating layers formed and that the MIP was successfully prepared. Furthermore, Figure 4 shows the SEM image of SiO_2_ and MIP with a mean diameter of 480 nm and 740 nm accordingly, which reveals that a thickness of 260 nm for the imprinted polymer layer coating on the surface of the SiO_2_. Additionally, the surface morphology of MIP does not change noticeably from that of NIP (Figure 4b). This finding offered strong support for the idea that the molecular imprinting process, rather than a change in surface appearance, was responsible for the unique binding properties between MIP and NIP.

#### 3.1.3. Standard BET Analysis

MIP was characterized by nitrogen adsorption–desorption analysis with BET method. Specific surface area, total pore volume and average pore diameter of MIP, NIP and SiO_2_ substrate are displayed in Table 1. It was evident that MIP appeared to have a much larger specific surface area and pore volume than SiO_2_ and NIP. The specific surface area and pore volume of MIP were 9.7 times and 11.5 times more than of NIP, respectively. It implied that the imprinting process enhanced the specific surface area and pore volume. The template imprints presented in the structure of MIP helped form the comparable cavities and porosity which is beneficial for adsorption [45]. As a result, the enhancement in surface area of the MIP exhibited that the sol-gel imprinted polymer layer on the surface of SiO_2_ contained molecular recognition sites for quercetin.

### 3.2. Adsorption Kinetics of MIP

**Figure 5:**
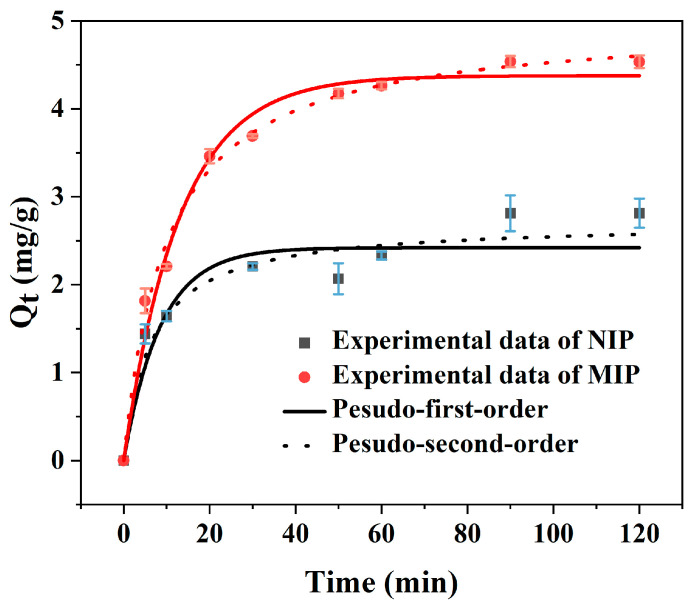
Adsorption kinetics of quercetin onto MIP and NIP by kinetics model simulation.

An essential element of the adsorption process is the adsorption rate. The relationship between the adsorption capacity (Q) and the time is depicted by the adsorption kinetics curve (see Figure 5). It was observed that as contact duration increased, quercetin adsorption increased on the MIP. During the first 30 min, the adsorption capacity increased rapidly and then slowly increased with time. Finally, the binding equilibrium almost reached at about 90 min for MIP. The adsorption trend of NIP was similar to MIP, but the adsorption capacity of NIP was relatively lower (Q_MIP_ = 4.53 mg/g and Q_NIP_ = 2.81 mg/g, IF = Q_MIP_/Q_NIP_ = 1.61). The reason why MIP achieved a higher quercetin adsorption capacity than NIP was that the imprinting cavities were formed on the surface of imprinted polymer layer and quercetin molecules can easily reach the specific recognition sites. The geometric shape affinity between quercetin molecules and quercetin imprinting cavities on the surface of MIP was thought to be the cause of the adsorption equilibrium. Adsorption cavities that were complementary to the template molecule quercetin in terms of both appearance and function were created through molecular imprinting. While there were no imprinted cavities for NIP and the adsorption process mainly was nonspecific. The outcomes demonstrated that the adsorption process was comparatively quick, which was helpful for saving time for the extraction procedure.

To investigate the mechanism of the adsorption process of MIP for quercetin, two mostly used kinetics models such as pseudo-first-order (Equation (4)) and pseudo-second-order kinetic model (Equation (5)) in their linearized forms were applied to analyze the experimental data.

(4) $$\ln\left(Q_{e}-Q_{t}\right)=lnQ_{e}-k_{1}t$$

(5) $$\frac{t}{Q_{t}}=\frac{1}{k_{2}Q_{e}^{2}}+\frac{t}{Q_{e}}$$

where Q_e_ and Q_t_ (mg/g) were the adsorption capacity at equilibrium condition and at time t (min), respectively. k_1_ (min^−1^) and k_2_ (g·mg^−1^·min^−1^) represented rate constants of pseudo-first-order and pseudo-second-order kinetic model, respectively.

Figure 5 shows the results of a nonlinear regression comparison of the kinetic models for quercetin adsorption by MIP and NIP, and Table 2 summarizes the related kinetic parameters and correlation coefficients. The degree to which the regression equation fits was shown by the correlation index R^2^. The regression values R^2^ for the pseudo-second-order models reached 0.9930, which was much greater than that of the pseudo-first-order models, as shown in Table 2. Additionally, the calculated adsorption capacity (Q_e,cal_ = 4.59 mg/g, Table 2) obtained from the pseudo-second-order model happened to coincide with the experimental adsorption capacity (Q_e,exp_ = 4.53 mg/g, Table 2). The findings implied that the kinetic process of the adsorption of quercetin on MIP was able to be described properly by the pseudo-second-order kinetic model.

### 3.3. Adsorption Isotherms of MIP

**Figure 6:**
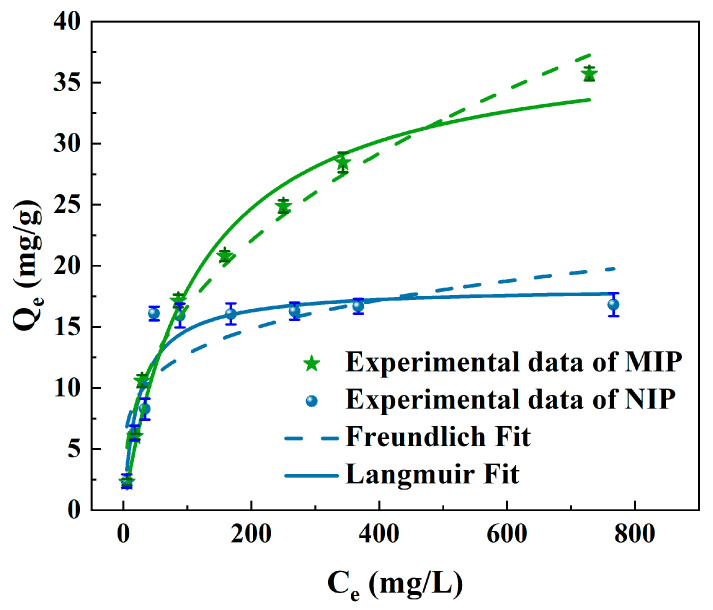
Adsorption isotherms of quercetin onto MIP and NIP by isotherms model simulation.

The maximal adsorption capacity was a significant factor of evaluating the sorbent efficiency. In Figure 6, varied initial concentrations were used to estimate the experimental equilibrium isotherms of MIP and NIP towards quercetin at room temperature. The adsorption capacity increased with increasing initial concentrations of quercetin, and the maximum static adsorption capacity attained 35.70 mg/g and 16.28 mg/g for MIP and NIP, respectively. A much higher adsorption capacity was reached on MIP compared to NIP (IF = Q_MIP_/Q_NIP_ = 2.19), demonstrating that MIP had specific adsorption and strong binding capacity for quercetin and the accessible imprinted sites embedded on the surface of MIP layer made quercetin easily entering into the imprinting cavities [46]. Due to a lack of imprinting cavities, and thus a lack of binding sites, the quercetin adsorption was smaller in NIP.

The equilibrium data were fitted using both the Langmuir and the Freundlich adsorption isotherm models in order to better understand the surface characteristics of MIP and NIP. The Langmuir model was better suited for a homogenous surface with monolayer adsorption, whereas the Freundlich model was suitable for a heterogeneous surface for multilayer adsorption. The goodness of fit was estimated by R^2^. Both the Langmuir model (Equation (6)) and the Freundlich model (Equation (7)) were expressed, respectively, as follows:

(6) $$Q_{e}=\frac{Q_{m}K_{L}C_{e}}{1+K_{L}C_{e}}$$

(7) $$Q_{e}=K_{F}C_{e}^{\frac{1}{n}}$$

where Q_e_ (mg·g^−1^) and C_e_ (mg·L^−1^) were the equilibrium adsorption capacity and the equilibrium concentration of quercetin in solution at adsorption equilibrium, respectively, Q_m_ (mg·g^−1^) was the maximum adsorption capacity of the MIP or NIP. The Langmuir constant, K_L_ (L·mg^−1^), was influenced by the binding sites’ affinities. The Freundlich constants K_F_ (mg·g^−1^) and n, which stand for the system’s adsorption capacity and adsorption favorability, respectively.

A comparison of the nonlinear regression plots fitted by the Langmuir and the Freundlich adsorption isotherm models for quercetin on MIP and is presented in Figure 6 and the parameters are listed in Table 3. The correlation coefficient R^2^ values of the Freundlich model was 0.9999, much higher than that of the Langmuir model. This means that the adsorption of quercetin on MIP could be better described by the Freundlich model. The results indicated that the surface of MIP was heterogeneous, which was in accordance with the characteristics of MIP. MIPs generally possess two types of interaction sites, specific sites and non-specific sites, which thus led to a heterogeneous population of sites with different affinities for the imprinted molecule in the MIP matrix [47]. Correspondingly, the Langmuir model (R^2^, 0.8888) gave a better fit than the Freundlich model (R^2^, 0.6742) for quercetin on NIP, which revealed that the adsorption of the quercetin molecule by NIP was primarily homogeneous and non-specific.

Table 4 compares the performance on the adsorption kinetics and isotherms of the MIP as a selective adsorbent for quercetin in our work with other quercetin-MIPs that previously reported by different methods in the literatures [30,32,48,49,50]. Apparently, the comparison studies revealed that MIP we prepared was excellent in terms of high adsorption capacity, short adsorption time, and fast adsorption kinetics.

### 3.4. Adsorption Specificity of the MIP

**Figure 7:**
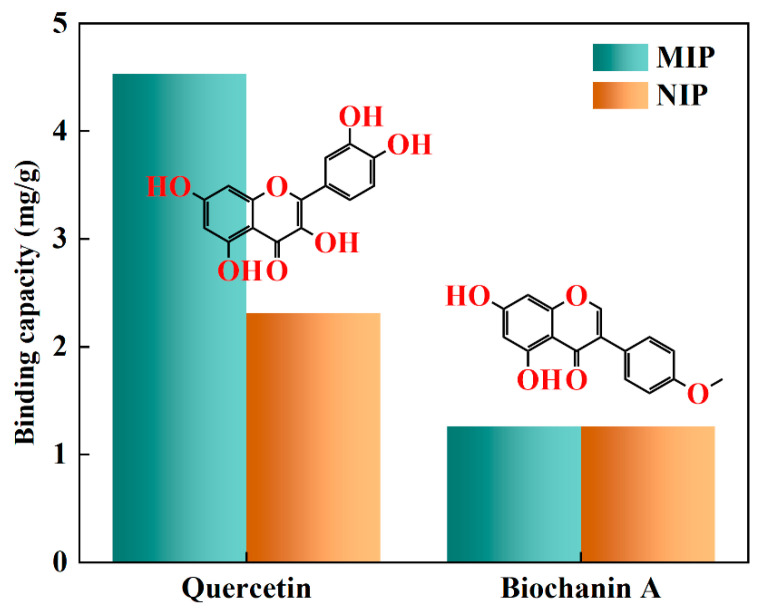
Selective adsorption of MIP and NIP.

To confirm the binding selectivity of MIP and NIP, biochanin A was chosen as the potential interferents due to its similar molecular structure with quercetin. Figure 7 shows the selective adsorption capacity of quercetin and its structural analogue biochanin A for MIP and NIP. As shown in Figure 7 and Table 5, obviously, MIP demonstrated a higher binding capacity to quercetin, which was about 3.60-fold of that to biochanin A (Q_MIP_ of quercetin was 4.53 mg/g, Q_MIP_ of biochanin A was 1.26 mg/g) because of its template-specific sites and higher affinity for the template quercetin. Despite being tiny enough to access the polymer layer on the surface of the support SiO_2_, the biochanin A molecule did not match the imprinted cavities and spatial arrangement of binding sites in the MIP. On the other hand, NIP showed lower adsorption capacity to quercetin than MIP and the same adsorption capacity to biochanin A, suggesting that NIP had no imprinted cavities and sites created during polymerization, bringing nonspecific interaction with quercetin and its reference compound biochanin A.

Table 5 also presents the imprinting factor (IF) and selectivity coefficient (α) for evaluating the selectivity of MIP towards quercetin and competitive compound. the value of IF shown in Table 5 for quercetin was 1.61, which is larger than that of biochanin A (IF = 1.00). The results revealed that the MIP had a high level of specificity for recognizing quercetin. Furthermore, the α values for quercetin, relative to biochanin A, was 1.61, meaning that the MIP had higher adsorption selectivity than that of the NIP. Consequently, it can be concluded that the MIP displayed remarkable specificity and great selectivity towards the template molecule quercetin.

### 3.5. Regeneration of MIP

**Figure 8:**
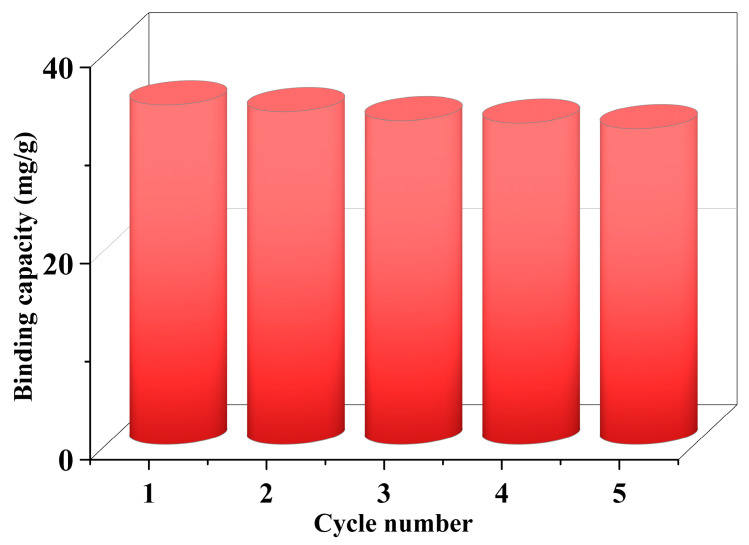
Reusability study of MIP.

Adsorbent regeneration is crucial in real-world industrial applications. In this study, the regeneration and the stability of MIP was investigated by measuring the binding capability of MIP for quercetin repeatedly after each adsorption–desorption cycle under the same experimental conditions. The adsorption capacity of MIP which was reused five times is given in Figure 8. It could be clearly seen that MIP had excellent stability with a negligible decrease in adsorption capability and the adsorption efficiency only lost about 7% at the fifth adsorption–regeneration cycle compared with the initial adsorption. It was logical to consider that MIP could be effectively regenerated for being reused at least five times.

## 4. Conclusions

In summary, a new type of sol-gel surface molecularly imprinted polymer with rough spherical morphology for template quercetin using SiO_2_ as the support, APTES as the functional monomer and TEOS as the cross-linker had been successfully employed to synthesize. The surface morphology and composition of the prepared MIP was investigated by SEM, FTIR, and nitrogen adsorption–desorption techniques. The results indicated that surface morphology of MIP was rough and the particle size of MIP and the support SiO_2_ was 480 nm and 740 nm accordingly, revealing that MIP layer with a thickness of 260 nm was successfully coated onto the surface of the SiO_2_. The N-H and C-H groups, respectively, were ascribed to the bands about 1499 cm^−1^ and 2932 cm^−1^ in the FTIR spectra of MIP. The outcomes showed that the MIP had been effectively manufactured. The specific surface area and pore volume of MIP were 52.10 m^2^ g^−1^ and 0.150 cm^3^ g^−1^, which were much larger than NIP and SiO_2_, implying that the imprinting process formed the comparable imprinted cavities and porosity, and thus enhanced the specific surface area and pore volume of MIP. Static binding experiments gave a result of adsorption equilibrium time within 90 min and the maximum adsorption capacity of 35.70 mg/g, suggesting MIP had a fast adsorption kinetic rate and high binding capacity for the template molecule quercetin. The pseudo-second-order kinetic model (R^2^ = 0.9930) and the Freundlich isotherm model (R^2^ = 0.9999) gave good fits to the data for the static adsorption data, respectively. Furthermore, the selectivity coefficient values for quercetin, relative to biochanin A, was 1.61, which displayed that MIP exhibited specific selectivity and satisfactory recognition capability towards template quercetin. The reusability of MIP was also outstanding that the binding capability of the MIP may be employed at least five times without noticeably deteriorating. All these results demonstrated that the potential application of the MIP was suitable for selective adsorption of quercetin. Additionally, it can be anticipated that it will be used to concentrate and purify trace analytes in the complex matrix.

## Figures and Tables

**Table 1:** BET surface area, pore volume and average pore diameter of the SiO_2_ support, NIP and MIP.

Samples	BET Surface Area (m^2^ g^−1^)	Pore Volume (cm^3^ g^−1^)	Average Pore Diameter (nm)
**SiO_2_**	10.35	0.023	1.19
**NIP**	5.39	0.013	1.19
**MIP**	52.10	0.150	1.19

**Table 2:** Kinetic parameters for the adsorption of quercetin onto MIP and NIP.

	Pseudo-First-Order				Pseudo-Second-Order		
Sample	Q_e,exp_ (mg·g^−1^)	Q_e,cal_ (mg·g^−1^)	k_1_ (min^−1^)	R^2^	Q_e,cal_ (mg·g^−1^)	k_2_ (mg·g·m in^−1^)	R^2^
MIP	4.53	4.37	0.07684	0.9828	4.59	0.01976	0.9930
NIP	2.81	2.42	0.1168	0.8718	2.57	0.05652	0.9321

**Table 3:** The parameters of the Langmuir and the Freundlich isotherm model for MIP and NIP.

	Langmuir				Freundlich		
Sample	Q_e,exp_ (mg·g^−1^)	Q_m_ (mg·g^−1^)	k_L_ (L·mg^−1^)	R^2^	k_F_ (mg·g^−1^)	n	R^2^
MIP	35.70	38.90	0.008682	0.9786	2.581	2.469	0.9999
NIP	16.28	28.27	0.04185	0.8888	4.793	4.690	0.6742

**Table 4:** Comparison of the data on the adsorption capacity and adsorption time with different quercetin-MIPs.

Adsorbents	Adsorption Capacity (mg·g^−1^)	Adsorption Time (min)	Ref
CMMs@MPS@MIPs	3.50	250	[48]
MIP	2.42	90	[32]
Fe_3_O_4_@MIPs	10.52	30	[30]
MIPs-8	0.52	50	[49]
DIPM-Qu	4.38	240	[50]
MIP	35.70	90	This work

**Table 5:** Adsorption selectivity parameters of MIP and NIP.

Adsorbates	Q_MIP_ (mg·g^−1^)	Q_NIP_ (mg·g^−1^)	IF	α
Quercetin	4.53	2.81	1.61	-
Biochanin A	1.26	1.26	1.00	1.61

## Data Availability

No new data were created.
